# Supplementary material for: Role and mechanism of NCAPD3 in promoting malignant behaviors in gastric cancer
Source: Front Pharmacol. 2024 Apr 22;15:1341039. doi: 10.3389/fphar.2024.1341039 (PMC11070777; doi:10.3389/fphar.2024.1341039)
Supplement: Supplementary file 11 [file DataSheet2.ZIP › GSEA/immunologic/my_analysis.Gsea.1599462003817/GSE18791_CTRL_VS_NEWCASTLE_VIRUS_DC_8H_UP.html]

Details for gene set GSE18791\_CTRL\_VS\_NEWCASTLE\_VIRUS\_DC\_8H\_UP[GSEA]

|  || Dataset | filtered\_dataset.sample\_info.cls#WT\_versus\_NCAPD3\_MUT |
| Phenotype | sample\_info.cls#WT\_versus\_NCAPD3\_MUT |
| Upregulated in class | WT |
| GeneSet | GSE18791\_CTRL\_VS\_NEWCASTLE\_VIRUS\_DC\_8H\_UP |
| Enrichment Score (ES) | 0.34797266 |
| Normalized Enrichment Score (NES) | 1.9887733 |
| Nominal p-value | 0.0031595577 |
| FDR q-value | 0.5851955 |
| FWER p-Value | 1.0 |
Table: GSEA Results Summary

  

Fig 1: Enrichment plot: GSE18791\_CTRL\_VS\_NEWCASTLE\_VIRUS\_DC\_8H\_UP      
 Profile of the Running ES Score & Positions of GeneSet Members on the Rank Ordered List

  

| SYMBOL | TITLE | RANK IN GENE LIST | RANK METRIC SCORE | RUNNING ES | CORE ENRICHMENT || 1 | 64172 | OSGEPL1 | 6 | 1.152 | 0.0431 | Yes |
| 2 | 84133 | ZNRF3 | 30 | 0.996 | 0.0674 | Yes |
| 3 | 222236 | NAPEPLD | 36 | 0.977 | 0.1040 | Yes |
| 4 | 22879 | MON1B | 151 | 0.735 | 0.0508 | Yes |
| 5 | 11162 | NUDT6 | 162 | 0.716 | 0.0730 | Yes |
| 6 | 57325 | CSRP2BP | 176 | 0.698 | 0.0923 | Yes |
| 7 | 51018 | RRP15 | 192 | 0.685 | 0.1095 | Yes |
| 8 | 55028 | C17orf80 | 222 | 0.659 | 0.1155 | Yes |
| 9 | 55172 | DNAAF2 | 224 | 0.657 | 0.1418 | Yes |
| 10 | 51250 | C6orf203 | 230 | 0.650 | 0.1650 | Yes |
| 11 | 84078 | KBTBD7 | 295 | 0.594 | 0.1426 | Yes |
| 12 | 81889 | FAHD1 | 299 | 0.592 | 0.1648 | Yes |
| 13 | 84640 | USP38 | 303 | 0.589 | 0.1869 | Yes |
| 14 | 147929 | ZNF565 | 311 | 0.587 | 0.2060 | Yes |
| 15 | 27079 | RPUSD2 | 320 | 0.582 | 0.2241 | Yes |
| 16 | 8125 | ANP32A | 326 | 0.578 | 0.2443 | Yes |
| 17 | 84135 | UTP15 | 330 | 0.576 | 0.2659 | Yes |
| 18 | 79896 | THNSL1 | 407 | 0.534 | 0.2322 | Yes |
| 19 | 25917 | THUMPD3 | 412 | 0.530 | 0.2511 | Yes |
| 20 | 5001 | ORC5 | 450 | 0.506 | 0.2449 | Yes |
| 21 | 200933 | FBXO45 | 456 | 0.500 | 0.2618 | Yes |
| 22 | 10940 | POP1 | 497 | 0.474 | 0.2521 | Yes |
| 23 | 29090 | TIMM21 | 537 | 0.453 | 0.2422 | Yes |
| 24 | 9416 | DDX23 | 553 | 0.445 | 0.2496 | Yes |
| 25 | 78988 | MRPL57 | 557 | 0.441 | 0.2656 | Yes |
| 26 | 9816 | URB2 | 581 | 0.434 | 0.2666 | Yes |
| 27 | 93134 | ZNF561 | 590 | 0.431 | 0.2785 | Yes |
| 28 | 9653 | HS2ST1 | 591 | 0.429 | 0.2962 | Yes |
| 29 | 55039 | TRMT12 | 599 | 0.424 | 0.3086 | Yes |
| 30 | 79697 | C14orf169 | 616 | 0.415 | 0.3140 | Yes |
| 31 | 55735 | DNAJC11 | 625 | 0.409 | 0.3250 | Yes |
| 32 | 51287 | COA4 | 660 | 0.396 | 0.3164 | Yes |
| 33 | 6427 | SRSF2 | 679 | 0.388 | 0.3192 | Yes |
| 34 | 56897 | WRNIP1 | 715 | 0.367 | 0.3087 | Yes |
| 35 | 83743 | GRWD1 | 720 | 0.364 | 0.3208 | Yes |
| 36 | 6182 | MRPL12 | 729 | 0.359 | 0.3297 | Yes |
| 37 | 134430 | WDR36 | 733 | 0.359 | 0.3423 | Yes |
| 38 | 27300 | ZNF544 | 759 | 0.343 | 0.3382 | Yes |
| 39 | 158 | ADSL | 796 | 0.326 | 0.3252 | Yes |
| 40 | 29960 | FTSJ2 | 805 | 0.319 | 0.3325 | Yes |
| 41 | 26608 | TBL2 | 821 | 0.304 | 0.3341 | Yes |
| 42 | 26121 | PRPF31 | 825 | 0.301 | 0.3443 | Yes |
| 43 | 10528 | NOP56 | 837 | 0.285 | 0.3480 | Yes |
| 44 | 23412 | COMMD3 | 894 | -0.312 | 0.3198 | No |
| 45 | 861 | RUNX1 | 1293 | -0.687 | 0.0566 | No |
| 46 | 55691 | FRMD4A | 1295 | -0.689 | 0.0842 | No |
Table: GSEA details [plain text format]

  

Fig 2: GSE18791\_CTRL\_VS\_NEWCASTLE\_VIRUS\_DC\_8H\_UP      
 Blue-Pink O' Gram in the Space of the Analyzed GeneSet

  

Fig 3: GSE18791\_CTRL\_VS\_NEWCASTLE\_VIRUS\_DC\_8H\_UP: Random ES distribution      
 Gene set null distribution of ES for **GSE18791\_CTRL\_VS\_NEWCASTLE\_VIRUS\_DC\_8H\_UP**
